# Fecal Methylmercury Correlates With Gut Microbiota Taxa in Pacific Walruses (*Odobenus rosmarus divergens*)

**DOI:** 10.3389/fmicb.2021.648685

**Published:** 2021-06-09

**Authors:** Sarah E. Rothenberg, Danielle N. Sweitzer, Bryna R. Rackerby, Claire E. Couch, Lesley A. Cohen, Heather M. Broughton, Sheanna M. Steingass, Brianna R. Beechler

**Affiliations:** ^1^College of Public Health and Human Sciences, Oregon State University, Corvallis, OR, United States; ^2^Carlson College of Veterinary Medicine, Oregon State University, Corvallis, OR, United States; ^3^Department of Food Science and Technology, College of Agricultural Sciences, Oregon State University, Corvallis, OR, United States; ^4^Department of Fisheries, Wildlife, and Conservation Sciences, College of Agricultural Sciences, Oregon State University, Corvallis, OR, United States; ^5^Department of Biology, Oregon State University-Cascades, Bend, OR, United States; ^6^Department of Fisheries, Wildlife, and Conservation Sciences, Marine Mammal Institute, Oregon State University, Corvallis, OR, United States

**Keywords:** marine mammal, metabolism, Arctic, pinniped, microbiome, colon, alpha diversity, stable isotopes of C and N

## Abstract

**Objectives:**

Methylmercury metabolism was investigated in Pacific walruses (*Odobenus rosmarus divergens*) from St. Lawrence Island, Alaska, United States.

**Methods:**

Total mercury and methylmercury concentrations were measured in fecal samples and paired colon samples (*n* = 16 walruses). Gut microbiota composition and diversity were determined using 16S rRNA gene sequencing. Associations between fecal and colon mercury and the 24 most prevalent gut microbiota taxa were investigated using linear models.

**Results:**

In fecal samples, the median values for total mercury, methylmercury, and %methylmercury (of total mercury) were 200 ng/g, 4.7 ng/g, and 2.5%, respectively, while in colon samples, the median values for the same parameters were 28 ng/g, 7.8 ng/g, and 26%, respectively. In fecal samples, methylmercury was negatively correlated with one *Bacteroides* genus, while members of the Oscillospirales order were positively correlated with both methylmercury and %methylmercury (of total mercury). In colon samples, %methylmercury (of total mercury) was negatively correlated with members of two genera, *Romboutsia* and *Paeniclostridium*.

**Conclusions:**

Median %methylmercury (of total mercury) was 10 times higher in the colon compared to the fecal samples, suggesting that methylmercury was able to pass through the colon into systemic circulation. Fecal total mercury and/or methylmercury concentrations in walruses were comparable to some human studies despite differences in seafood consumption rates, suggesting that walruses excreted less mercury. There are no members (at this time) of the Oscillospirales order which are known to contain the genes to methylate mercury, suggesting the source of methylmercury in the gut was from diet and not *in vivo* methylation.

## Introduction

Mercury (Hg) is a global pollutant, which has contaminated the Arctic food web despite the lack of nearby anthropogenic Hg point sources (Arctic Monitoring and Assessment Program (AMAP), 2011). Hg is transported to the Arctic by air currents and ocean currents and through river runoff (Arctic Monitoring and Assessment Program (AMAP), 2011). In aquatic environments, anaerobic microorganisms convert less toxic inorganic Hg(II) (IHg) to methylmercury (MeHg), a known neurotoxin (Parks et al., 2013). MeHg is degraded and re-volatilized into the atmosphere (Sellers et al., 1996; Marvin-DiPasquale and Oremland, 1998; Barkay et al., 2003) or biomagnified in the aquatic food web (Arctic Monitoring and Assessment Program (AMAP), 2011). Highest MeHg concentrations are found in the tissues of apex predators, including Arctic marine mammals (Den et al., 2006; Arctic Monitoring and Assessment Program (AMAP), 2011; Dietz et al., 2013; Rea et al., 2020). This is important for Arctic indigenous communities, which rely on marine foods as part of their traditional diet (Arctic Monitoring and Assessment Program (AMAP), 2011).

Metabolism of MeHg is mediated in part by gut microbiota, mainly through MeHg demethylation (i.e., degradation) (Rowland et al., 1975, 1984); however, many knowledge gaps remain concerning how gut microbes may impact the speciation, absorption, or excretion of Hg. In seafood, MeHg typically comprises >95% of total mercury (THg) (=MeHg + IHg) (Bloom, 1992). Conversely, in human fecal samples, IHg comprises a far higher percentage of THg (up to 100%) (Ishihara, 2000; Rand et al., 2016; Rothenberg et al., 2016, 2019; Caito et al., 2018; Rand and Caito, 2019), which is thought to reflect demethylation of MeHg by gut microbiota (Rowland et al., 1984). The intestinal epithelium is considered a barrier to IHg, more so than MeHg (Seko et al., 1981; Vázquez et al., 2013); thus, MeHg demethylation in the gut may reduce the reabsorption of MeHg into the host’s circulation. MeHg demethylation may also occur in the liver due to the formation of Hg-selenium complexes (Caurant et al., 1996; Lailson-Brito et al., 2012; Manhães et al., 2021); however, in a marine fish (black seabream, *Acanthopagrus schlegeli*), the rate of MeHg demethylation in the liver was on average 600 times slower compared to the intestine, suggesting that the intestine was the predominant site for MeHg demethylation in some species (Wang et al., 2017).

To date, one commensal methanogen (*Methanomassiliicoccus luminyensis*) isolated from human feces contained the gene cluster (*hgcA* and *hgcB*) required for microbial IHg methylation (Parks et al., 2013). However, organisms encoding *hgcA* and *hgcB*, including *M. luminyensis*, have not been found in human fecal samples (Podar et al., 2015; Rothenberg et al., 2016), suggesting the main source of MeHg was from diet, not microbial IHg methylation.

Prior studies concerning MeHg metabolism have focused on human populations (Rand et al., 2016; Rothenberg et al., 2016, 2019; Caito et al., 2018) or experimental animal models (Bridges et al., 2018; Zhang et al., 2019; Lin et al., 2020), while few, if any, studies have focused on fish-consuming wildlife in natural settings. As a large carnivorous marine mammal, the Pacific walrus (*Odobenus rosmarus divergens*) is a reservoir for global contaminants, including MeHg (Arctic Monitoring and Assessment Program (AMAP), 2011, 2018; Braune et al., 2015; Dietz et al., 2013; Quakenbush et al., 2016). In this study, associations between MeHg and gut microbiota were investigated in wild Pacific walruses. We hypothesize that associations with gut microbiota will differ between organic and inorganic Hg species. We also hypothesize that the colon, including the epithelium and mucosal regions, will accumulate more MeHg than IHg, potentially reflecting greater permeability of MeHg (Seko et al., 1981; Vázquez et al., 2013). This is an ancillary study to a larger study investigating immunity and parasite burden in the Pacific walrus.

## Materials and Methods

### Site Description and Tissue Collection

**FIGURE 1:**
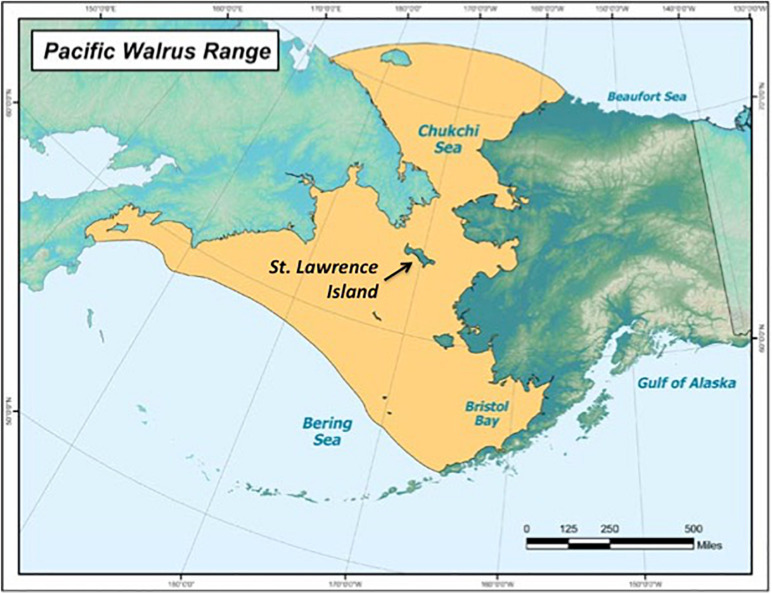
The Pacific walrus (*Odobenus rosmarus divergens*) range, including St. Lawrence Island, Alaska, which lies in the migration path (source: Alaska Department of Fish and Game (ADFG), 2020).

The Pacific walrus spends most of its life in the Arctic and subarctic regions, between the northern Bering and Chukchi Seas (Alaska Department of Fish and Game (ADFG), 2020). St. Lawrence Island, Alaska, United States (latitude: 63.4090, longitude: –170.3929), lies in the migration pathway, which walruses use as a haul-out site for resting (Figure 1; Alaska Department of Fish and Game (ADFG), 2020). St. Lawrence Island has approximately 1,400 Yupik residents who live in two neighboring Native Village, Gambell and Savoonga, and rely heavily on Pacific walruses as a food source (Quakenbush et al., 2016). The Native Village of Gambell is on the island’s northwest cape, while the Native Village of Savoonga is located on the northern coast, 63 km southeast of Gambell.

During the 2019 spring subsistence harvest (April–June), tissue samples (including the distal colon) from 37 walruses were collected by hunters and obtained for analysis through agreements with the Native Village of Savoonga and Gambell, the Eskimo Walrus Commission, and the US Fish and Wildlife Service (Gambell: 8 walruses; Savoonga: 29 walruses). Frozen tissue samples were transferred to Oregon State University for sample analysis under a letter of authorization from the US Fish and Wildlife Service to co-authors HB, SS, BB, and SR. Local hunters determined the sex and age classification (adult or subadult). Walruses were considered subadults if the tusk length was <30.5 cm (<12 in.) for males or <20.3 cm (<8 in.) for females; otherwise, walruses were considered adults (Quakenbush et al., 2016). Frozen (-20°C) fecal samples (homogenized) and colon sections were transferred into polypropylene vials or plastic Ziplock bags and stored at –80°C. For this analysis, paired fecal and colon samples were available for 16 walruses, and paired vibrissae (whiskers) were available for 13 walruses.

### Mercury Analyses

THg and MeHg concentrations were analyzed in fecal and colon samples at the Oregon State University Mercury Lab. The IHg concentration was estimated by subtraction (THg - MeHg).

Porcelain cups (250 ml) were acid-washed overnight in 1.2 N hydrochloric acid, triple-rinsed with Milli-Q H_2_O, and air-dried between each use. Prior to analysis, the colon sections were sonicated in 1% Triton X-100 (v/v) for 15 min, rinsed with Milli-Q H_2_O, and air-dried in acid-washed porcelain cups. The MeHg concentrations in feces and colon sections were analyzed using solvent extraction (Bloom et al., 1997). Briefly, frozen fecal samples (∼0.8 g) or air-dried colon samples (∼0.2 g) were weighed into 50-ml polypropylene tubes, and the samples were leached for 1 h in 5 ml of 18% (w/v) potassium bromide + 5% (v/v) sulfuric acid and 1 ml of 1 M copper sulfate solution. Then, 10 ml of dichloromethane (CH_2_Cl_2_) was added; the samples were shaken for 1 h and centrifuged (3,000 *g* for 30 min), and the phases were separated (Whatman 1-PS). The CH_2_Cl_2_ layer was evaporated by heating vials in a water bath (60–70°C) for 1.5 h, and the final volume was raised to 40 ml using Milli-Q H_2_O. The MeHg concentrations were quantified by gas chromatography–cold vapor atomic fluorescence spectrometry, according to US Environmental Protection Agency (US EPA) Method 1630 (U.S. Environmental Protection Agency, 2001), using sodium tetraethylborate as the derivatizing agent (Model-III Detector, Brooks Rand Instruments, Seattle, WA, United States). The THg concentrations in fecal and colon samples were measured directly without digestion by thermal decomposition, amalgamation, and atomic absorption spectrophotometry, following US EPA Method 7473 (U.S. Environmental Protection Agency, 2007) (Lumex, Model RA-915+/PYRO-915+, St. Petersburg, Russia).

The THg and MeHg concentrations in air-dried colon sections were reported in dry weight. The THg and MeHg concentrations in fecal samples were converted from wet weight to dry weight, after drying an aliquot (average mass: 0.09 g) at 105°C overnight. Hg analyses were completed for fecal samples and colon sections within 3 and 21 months, respectively, after the walruses were harvested.

### Mercury Quality Assurance/Quality Control

For THg and MeHg, the recovery of standard reference materials and matrix spikes averaged 88–99% and 82–95%, respectively (Supplementary Table 1). The relative standard deviation between replicate analyses for THg and MeHg averaged 20% and 17%, respectively (THg: *n* = 23 replicates, MeHg: *n* = 7 replicates). There was insufficient mass of remaining fecal sample to analyze replicates for the wet/dry ratio.

### Stable Isotopes of δ^15^N and δ^13^C in Walrus Vibrissae (Whiskers)

Stable isotopes of nitrogen (δ^15^N) and carbon (δ^13^C) in marine mammal whiskers have been used to infer diet (trophic position) and potential foraging habitat (geographic origin), respectively (Hobson et al., 1996; Seymour et al., 2014; Rea et al., 2020). Stable isotopes in walrus whiskers were analyzed at Oregon State University’s Stable Isotope Laboratory, in the proximal 1-cm whisker segment, representing the most recent exposure. Using an estimated growth rate of 0.1–0.2 mm/day for the Pacific walrus (Seymour et al., 2014), this whisker length corresponded to approximately 3.3–6.6 months. The whiskers were washed using 2-mercaptoethanol, triple-rinsed with Milli-Q H_2_O, and air-dried. The samples were flash-combusted at >1,000°C using a Carlo Erba NA1500 elemental analyzer. Carbon and nitrogen were analyzed using a DeltaPlus XL isotope ratio mass spectrometer. Differences (δ) of ^13^C/^12^C and ^15^N/^14^N were measured relative to Vienna Pee Dee Belemnite and atmospheric nitrogen (N_2_), respectively, using the following equation:

(1) $$\delta X=\left[\left(\frac{R_{\text{sample}}}{R_{\text{standard}}}\right)-1\right]\times 10^{3}$$

where δ*X* refers to δ^15^N or δ^13^*C*, and *R*_*sample*_, and *R*_*standard*_ refer to the relative difference between sample and standard isotope ratios, respectively. Results are reported as per mil (‰) deviation with a precision of ± 0.2‰ for δ^15^N and ± 0.1‰ for δ^13^C.

### PCR Sequencing and Processing

Genomic DNA was extracted using Qiagen PowerSoil Pro Kit, following the manufacturer’s instructions. An incubation step was added (65°C for 10 min) to improve the lysis of recalcitrant taxa^1^, just prior to bead disruption (max speed, 10 min; OMNI Bead Ruptor 24). Extracted DNA was analyzed at the Oregon State University Center for Genome Research and Biocomputing, following the standard Earth Microbiome protocol for 16s rRNA sequencing^2^ (Caporaso et al., 2011, 2012). Briefly, we amplified a 450-bp region of the V4 region of the bacterial 16S gene. The extracted DNA was subjected to a first round of 16S PCR amplification using the updated 515F forward primer (Parada et al., 2016) and 806R reverse primer (Apprill et al., 2015) (forward primer, 5′-GTGYCAGCMGCCGCGGTAA-3′; reverse primer, 5′-GGACTACNVGGGTWTCTAAT-3′). Amplicons were cleaned, indexed, and normalized prior to sequencing on the Illumina MiSeq platform, resulting in 250-bp paired-end reads.

Amplicon sequence variants (ASVs) were inferred using the DADA2 pipeline (version 1.14.1) (Callahan et al., 2016) (see Supplementary Material for details and Supplementary Table 2). Full-length 16S ribosomal RNA sequences were downloaded from the All Species Living Tree Project (SILVA, version 138) and aligned to ASVs. A sterile water sample was included as negative control to account for the effects of possible sample handling contaminants. Any ASVs observed within negative control were subsequently removed from all samples, resulting in a total of 344 ASVs. Alpha diversity indices were determined using the R package “vegan” (version 2.5-7), including the observed number of ASVs, Shannon’s diversity index, Simpson’s diversity index, and Pielou’s measure of evenness. Consistent with proposed nomenclature changes, the phylum Bacteroidetes has been re-named Bacteroidota (Whitman et al., 2018).

### Statistics and Bioinformatics

Associations between Hg [THg, MeHg, IHg, and %MeHg (of THg)] and walrus sampling location and age class were investigated using Wilcoxon rank sum test. Hg species in fecal and colon samples were compared using paired *t*-tests (two-tailed). Correlations between Hg species, stable isotopes of nitrogen (δ^13^N) and carbon (δ^13^C), and alpha diversity indices were determined using Spearman’s correlation.

To assess the associations between fecal and colon Hg species and gut microbiota taxa, we employed Microbiome Multivariable Association with Linear Models (Maaslin2) in R (Mallick et al., 2020). Because of the limited sample size (*n* = 16 samples), ASVs were included if measured in at least 80% of walrus gut microbiota samples, reducing the number of ASVs from 344 to 24. These 24 ASVs represented the most prevalent gut microbiota taxa. ASVs were normalized using relative abundance and transformed using the default transformation in Maaslin2 (arcsine-square-root transformation), which has been applied to proportional data (Sokal and Rohlf, 2012). A log_10_ transformation was applied to Hg variables, which were right-skewed.

Using Wilcoxon rank sum test or Spearman’s correlation, a *p*-value of 0.05 was used as a guide for significance. Using Maaslin2, *q*-values (false discovery rate-corrected *p*-values) were calculated (Mallick et al., 2020), and *q* ≤0.25 was used as a guide for significance. Statistical analyses were performed using Stata (Version 9.2, College Station, TX, United States) and the R-platform (Version 4.0.2, 06 June 2020) (R Core Team, 2020).

## Results

### Walrus Summary Data

The majority of walruses (75%) included in this study were harvested by hunters in Savoonga, compared to Gambell (Table 1). All walruses were male, while 56% of the walruses were classified as adults, 19% were classified as subadults, and the remaining 25% were not classified.

**TABLE 1:** Associations between walrus characteristics and mercury concentrations in fecal samples (*n* = 16 walruses).

	*N* (%)	THg (ng/g dw) Median (range)	*p*-value	MeHg (ng/g dw) Median (range)	*p*-value	IHg^*a*^ (ng/g dw) Median (range)	*p*-value	%MeHg (of THg) Median (range)	*p*-value
All	16 (100)	200 (72, 650)	NA	4.7 (0.96, 70)	NA	190 (71, 340)	NA	2.5 (0.46, 30)	NA
**Site**									
Savoonga	12 (75)	190 (72, 400)	0.07	4.7 (0.96, 70)	0.72	180 (71, 340)	0.04*	2.5 (1.1, 30)	0.63
Gambell	4 (25)	420 (154, 650)		6.2 (3.0, 26)		400 (150, 650)		2.4 (0.46, 5.8)	
**Age**									
Adult	9 (56)	200 (72, 650)	0.64	4.9 (1.4, 70)	0.93	180 (71, 650)	0.41	2.8 (0.46, 30)	0.78
Subadult	3 (19)	210 (170, 450)		4.5 (2.0, 26)		200 (170, 420)		2.2 (1.1, 5.8)	
Missing	4 (25)	200 (81, 400)		6.2 (0.96, 17)		190 (80, 390)		2.4 (1.2, 7.2)	

*p < 0.05. The *p*-values are for Wilcoxon rank sum test for non-missing categories. dw, dry weight; IHg, inorganic mercury(II); MeHg, methylmercury; THg, total mercury. ^*a*^IHg = THg – MeHg.

### Mercury Species in Fecal and Colon Samples

For all walruses (Table 1), the median fecal THg and MeHg concentrations were 200 ng/g (THg range: 72–650 ng/g) and 4.7 ng/g (MeHg range: 0.96–70 ng/g), respectively, while the median %MeHg (of THg) was 2.5% [%MeHg (of THg) range: 0.46–30%]. The walruses harvested by hunters in Gambell had higher fecal IHg concentrations compared to the walruses harvested by hunters in Savoonga, although the reason was uncertain. No differences in Hg species in fecal samples were observed between adults and subadults.

The distribution of Hg species in colon samples (Table 2) differed from fecal samples (Table 1). The median colon THg and MeHg concentrations were 28 ng/g (THg range: 15–95 ng/g) and 7.8 ng/g (MeHg range: 3.3–57 ng/g), respectively, while the median %MeHg (of THg) was 26% [%MeHg (of THg) range: 16–60%]. Compared to fecal samples, the median %MeHg (of THg) in colon tissue was 10 times higher (26 vs. 2.5%). Using paired fecal and colon samples, the MeHg concentrations did not differ; however, the THg and IHg concentrations were higher in fecal samples, while %MeHg (of THg) was higher in colon sections (two-tailed *t*-test, *p* < 0.0001 for all) (when Hg variables were log_10_-transformed). No differences were observed between colon Hg species and harvest site (i.e., the Native Village of Gambell and Savoonga); however, %MeHg (of THg) was higher in adults compared to subadults (Table 2).

**TABLE 2:** Associations between walrus characteristics and mercury concentrations in colon samples (*n* = 16 walruses).

	*N* (%)	THg (ng/g dw) Median (range)	*p*-value	MeHg (ng/g dw) Median (range)	*p*-value	IHg^*a*^ (ng/g dw) Median (range)	*p*-value	%MeHg (of THg) Median (range)	*p*-value
All	16 (100)	28 (15, 95)	NA	7.8 (3.3, 57)	NA	20 (8.8, 59)	NA	26 (16, 60)	NA
**Site**									
Savoonga	12 (75)	27 (15, 95)	0.33	7.8 (3.3, 57)	1.0	20 (8.8, 59)	0.23	27 (18, 60)	0.11
Gambell	4 (25)	42 (21, 60)		7.4 (5.9, 12)		35 (15, 49)		19 (16, 29)	
**Age**									
Adult	9 (56)	27 (17, 95)	0.93	7.9 (6.3, 57)	0.93	20 (8.8, 59)	0.78	29 (19, 60)	0.052
Subadult	3 (19)	38 (18, 47)		8.5 (3.3, 10)		27 (15, 39)		18 (18, 27)	
Missing	4 (25)	29 (15, 60)		5.3 (3.9, 12)		23 (11, 49)		21 (16, 26)	

*The *p*-values are for Wilcoxon rank sum test for non-missing categories. IHg, inorganic mercury(II); MeHg, methylmercury; THg, total mercury. ^*a*^IHg = THg – MeHg.*

In both fecal and colon samples, positive associations were observed between the concentrations of THg and MeHg, THg and IHg, and MeHg and IHg (Table 3). However, the correlation between MeHg and %MeHg (of THg) was stronger in fecal samples (Spearman’s rho: 0.87) compared to colon sections (Spearman’s rho: 0.26). In addition, the direction of association between THg and %MeHg (of THg) differed between fecal and colon samples. In colon sections, a negative association was observed (Spearman’s rho: –0.17), while in fecal samples a positive association was observed (Spearman’s rho: 0.31). Likewise, differences in the direction of association were observed between IHg and %MeHg (of THg) (Spearman’s rho: colon –0.40, fecal 0.18).

**TABLE 3:** Spearman’s correlation for mercury species (*n* = 16 walruses).

	THg	MeHg	IHg^*a*^	%MeHg (of THg)
**Fecal samples**				
THg	1			
MeHg	0.69**	1		
IHg^*a*^	0.97***	0.58*	1	
%MeHg (of THg)	0.31	0.87***	0.18	1
**Colon samples**				
THg	1			
MeHg	0.81***	1		
IHg^*a*^	0.93***	0.70**	1	
%MeHg (of THg)	–0.17	0.26	-0.40*	1

*p < 0.05, **p < 0.01, ***p < 0.001. IHg, inorganic mercury(II); MeHg, methylmercury; THg, total mercury. ^*a*^IHg = THg – MeHg.

### Whisker δ^13^C and δ^15^N and Fecal Mercury Species

For δ^13^C, the average ± 1SD was –15.7 ± 0.45‰ (range: –16.3 to –14.5‰), and for δ^15^N, the average ± 1SD was 13.6 ± 0.83‰ (range: 12.4 to 15.7‰). Associations between fecal THg, MeHg, IHg, and %MeHg (of THg) and stable isotopes of δ^15^N were positive; however, all correlation coefficients were non-significant (Spearman’s rho range: 0.12–0.29). Similarly, associations between fecal THg, MeHg, IHg, and %MeHg (of THg) and stable isotopes of δ^13^C were positive but non-significant (Spearman’s rho range: 0.15–0.35).

### Gut Microbiota Alpha Diversity Indices and Mercury Species

**FIGURE 2:**
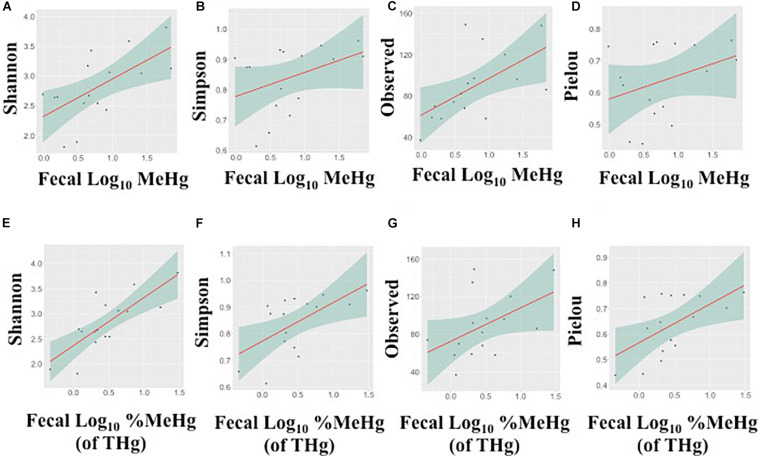
Associations between the alpha diversity indices and fecal methylmercury (MeHg) **(A–D)** or %MeHg [of total mercury (THg)] **(E–H)** (both log_10_-transformed), including **(A,E)** Shannon’s diversity index, **(B,F)** Simpson’s diversity index, **(C,G)** the observed number of amplicon sequence variants, and **(D,H)** Pielou’s measure of evenness. Each plot includes the linear regression line ± standard error of the regression line.

In fecal samples, MeHg was strongly positively correlated with the observed number of ASVs and Shannon’s diversity index (Spearman’s rho range: 0.54–0.63), while fecal %MeHg (of THg) was strongly positively correlated with all four indices, including the observed number of ASVs, Shannon’s diversity index, Simpson’s diversity index, and Pielou’s measure of evenness (Spearman’s rho range: 0.50–0.71) (Figure 2). The trends between the observed number of ASVs and fecal THg and IHg were also positive, although non-significant (Spearman’s rho range: 0.45–0.48). In colon samples, there were no significant associations between Hg species and alpha diversity indices. Likewise, no differences in alpha diversity indices were observed between the two harvest sites, i.e., the Native Village of Gambell and Savoonga, or between adult and subadult walruses.

### Gut Microbiota and Mercury Species

Twenty-four ASVs met our inclusion criteria for Maaslin2 analysis, which comprised 22–83% relative abundance (median: 56% relative abundance) (Supplementary Table 3 and Supplementary Figure 1). ASVs belonged to three phyla (Firmicutes, Bacteroidota, and Fusobacteriota), four classes (Bacteroidia, Bacilli, Clostridia, and Fusobacteria), and seven orders (Bacteroidales, Clostridiales, Erysipelotrichales, Fusobacteriales, Lachnospirales, Oscillospirales, and Peptostreptococcales-Tissierellales). The most abundant ASVs (average ± SD) included a *Fusobacterium* genus (family *Fusobacteriaceae*; 12 ± 19% relative abundance), a *Paeniclostridium* genus (family *Peptostreptococcaceae*; 9.1 ± 10% relative abundance), and a member of the species *Clostridium sensu stricto* cluster 1 *perfringens* (family *Clostridiaceae*; 6.5 ± 13% relative abundance). As noted in the section “Introduction,” *M. luminyensis* contains the gene cluster (*hgcA* and *hgcB*) required for microbial IHg methylation (Parks et al., 2013). However, among all 344 ASVs, members of the genus *Methanomassiliicoccus* were not observed.

Results from the Maaslin2 linear models are presented in Supplementary Tables 4–7 (for fecal samples) and Supplementary Tables 8–11 (for colon samples).

**FIGURE 3:**
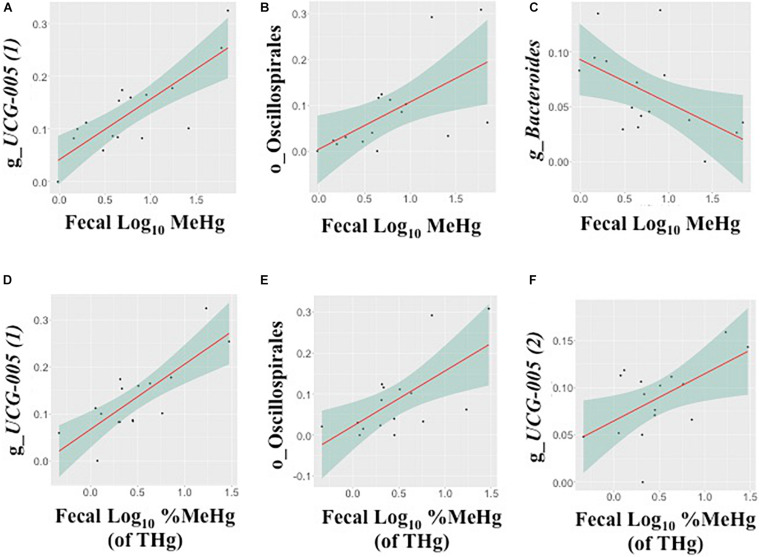
Associations between the relative abundance of gut microbiota amplicon sequence variants (ASVs, arcsine-square root transformed) and fecal methylmercury (MeHg) **(A–C)** or %MeHg [of total mercury (THg)] **(D–F)** (both log_10_-transformed), including **(A)** genus *UCG-005* (1) (family: *Oscillospiraceae*), **(B)** order Oscillospirales, **(C)** genus *Bacteroides* (family: *Bacteroidaceae*), **(D)** genus *UCG-005* (1) (family: *Oscillospiraceae*), **(E)** order Oscillospirales, and **(F)** genus *UCG-005 (2)* (family: *Oscillospiraceae*) (*n* = 16 walruses). Each plot includes the linear regression line ± standard error of the regression line (see Table 4).

**FIGURE 4:**
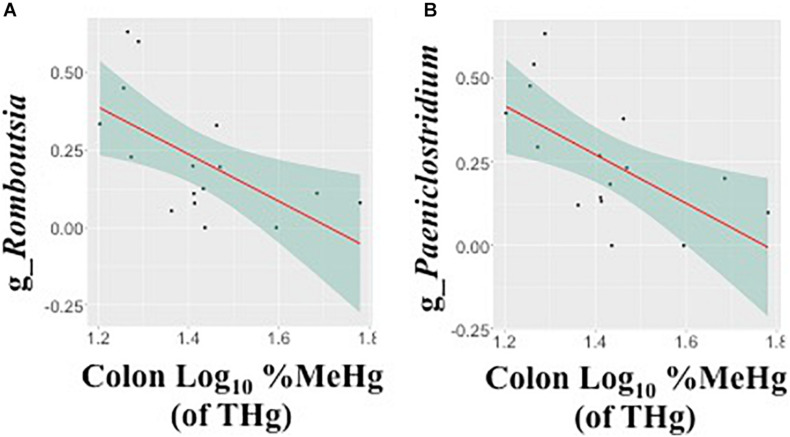
Associations between the relative abundance of gut microbiota amplicon sequence variants (arcsine-square root transformed) and colon %methylmercury (MeHg) [of total mercury (THg)] (log_10_-transformed), including **(A)** genus *Romboutsia* (family: *Peptostreptococcaceae*) and **(B)** genus *Paeniclostridium* (family: *Peptostreptococcaceae*) (*n* = 16 walruses). Each plot includes the linear regression line ± standard error of the regression line (see Table 4).

In fecal samples, log_10_ MeHg and log_10_ %MeHg (of THg) were each correlated (positive and negative) with three ASVs (Table 4, Figure 3, and Supplementary Tables 6, 7). ASVs correlated with fecal log_10_ MeHg included a member of the *Bacteroides* genus (negatively correlated), a member of the genus UCG-005 (family: *Oscillospiraceae*; positively correlated), and an unclassified member of the Oscillospirales order (positively correlated). The latter two ASVs were also positively correlated with fecal log_10_ %MeHg (of THg). In addition, a second member of the genus UCG-005 (family: *Oscillospiraceae*) was positively correlated with fecal log_10_ %MeHg (of THg). In colon samples, log_10_ %MeHg (of THg) was negatively correlated with two ASVs, including members of the *Romboutsia* genus and the *Paeniclostridium* genus (Table 4, Figure 4, and Supplementary Tables 10, 11).

**TABLE 4:** Associations between mercury species (log_10_-transformed) and gut microbiota amplicon sequence variants (arcsine-square root transformed) using Microbiome Multivariable Association with Linear Models (Maaslin2) (*n* = 16 walruses).

Phylum	Class	Order	Family	Genus	Beta coefficient	Number of samples not 0	*p*-value	*q*-value (FDR adjusted *p*-value)
**Log_10_ methylmercury in feces**								
Firmicutes	Clostridia	Oscillospirales	*Oscillospiraceae*	*UCG-005 (1)*	0.11	15	0.0002	0.004
Firmicutes	Clostridia	Oscillospirales	NA	NA	0.10	14	0.01	0.16
Bacteroidota	Bacteroidia	Bacteroidales	*Bacteroidaceae*	*Bacteroides*	–0.04	15	0.03	0.22
**Log_10_ %methylmercury (of total mercury) in feces**								
Firmicutes	Clostridia	Oscillospirales	*Oscillospiraceae*	*UCG-005 (1)*	0.14	15	0.0002	0.005
Firmicutes	Clostridia	Oscillospirales	NA	NA	0.13	14	0.007	0.08
Firmicutes	Clostridia	Oscillospirales	*Oscillospiraceae*	*UCG-005 (2)*	0.05	15	0.02	0.18
**Log_10_ %methylmercury (of total mercury) in colon**								
Firmicutes	Clostridia	Peptostreptococcales-Tissierellales	*Peptostreptococcaceae*	*Romboutsia*	–0.76	14	0.01	0.16
Firmicutes	Clostridia	Peptostreptococcales-Tissierellales	*Peptostreptococcaceae*	*Paeniclostridium*	–0.73	14	0.01	0.16

*FDR, false discovery rate.*

There were no ASVs that were correlated (positive or negative) with log_10_ THg or log_10_ IHg in fecal or colon samples (Supplementary Tables 4, 5, 8, 9).

## Discussion

The results suggested that dietary MeHg was microbially mediated in the walrus gut, similar to human studies (Rand et al., 2016; Rothenberg et al., 2016, 2019; Caito et al., 2018; Guo et al., 2018). In fecal and colon samples, MeHg and %MeHg (of THg) were more strongly correlated (positive and negative) with gut microbiota taxa compared to THg and IHg. Additionally, in fecal samples, MeHg and %MeHg (of THg) were more strongly correlated with within-walrus alpha diversity indices compared to THg and IHg.

### Associations Between Colon MeHg and Gut Microbiota ASVs

In walruses, the median %MeHg (of THg) was 10 times higher in the colon compared to fecal samples, suggesting that MeHg was able to pass through the colon into the systemic circulation more so than IHg. In colon samples, %MeHg (of THg) was negatively correlated with two genera, *Romboutsia* and *Paeniclostridium. Romboutsia* spp. are dependent on mucus-degrading microbes for host-derived carbohydrates (Gerritsen et al., 2017), and thus changes in mucosa production may alter *Romboutsia* abundance. In human studies, the genus *Romboutsia* was depleted more than sixfold in colonic mucosa (with polyps) compared to healthy marginal tissue (Mangifesta et al., 2018). Similarly, in a separate human study, *Paeniclostridium* spp. were more depleted in colonic tumor mucosa tissues compared with the adjacent normal mucosa tissues (Wang et al., 2020). Both studies suggested that depletion of *Romboutsia* and *Paeniclostridium* spp. may indicate alterations of the mucosa (Mangifesta et al., 2018; Wang et al., 2020). However, among commercial hens, intestinal permeability (measured using the fluorescein isothiocyanate-dextran assay) was negatively associated with *Romboutsia* spp. in cage-free hens and positively associated in conventional caged hens (Wiersema et al., 2021). The differences were potentially due to the different *Romboutsia* strains found in both types of hens (Wiersema et al., 2021). A lower relative abundance of both genera in the walrus gut possibly reflected changes in the mucosa, which contributed to a higher proportion of MeHg in the colon tissue.

### Variability in MeHg Metabolism vs. Differences in Prey Trophic Level

In walrus fecal samples, %MeHg (of THg) ranged from <1 to 30%, suggesting variability in the metabolism of MeHg or differences in prey trophic level. Whisker δ^15^N varied by 3.3‰ (range: 12.4–15.7‰), suggesting an increase of one trophic level in prey (Hobson et al., 1996). Positive associations have been observed between δ^15^N and THg or MeHg in Arctic food webs (Aubail et al., 2011; Clayden et al., 2015); however, in walrus fecal samples, the associations between δ^15^N and Hg parameters, including %MeHg (of THg), were non-significant. The results suggested that variability in MeHg metabolism, more so than differences in prey trophic level, potentially contributed to the wide range of values observed for %MeHg (of THg).

### Potential MeHg Degradation in the Walrus Gut

In the walrus gut, a weak positive correlation was observed between fecal THg and %MeHg (of THg) (Spearman’s rho: 0.31) (Table 3). Conversely, among human pregnant mothers and their neonates, a strong inverse correlation was observed in maternal fecal samples and meconium (Spearman’s rho range: –0.75 to –0.42, *n* = 14–28) (Rothenberg et al., 2016, 2019). An inverse association between THg and %MeHg (of THg) is thought to reflect microbial MeHg degradation. In natural surface waters (lakes, rivers, wetlands, and estuaries), an inverse trend between THg and %MeHg (of THg) was attributed to microbial MeHg degradation using the mer pathway, which involves MerA, the mercuric reductase, and MerB, the organomercurial lyase (Barkay et al., 2003; Schaefer et al., 2004). Although an inverse trend between THg and %MeHg (of THg) was observed in fecal samples among human pregnant mothers, a low abundance of *merA* was present in all fecal samples, while *merB* was lacking in all fecal samples (Rothenberg et al., 2016). These results suggested that other MeHg degradation pathways were potentially more important in the human gut, such as phagocytosis or oxidative demethylation (Rothenberg et al., 2016). In walrus fecal samples, a weak positive correlation between THg and %MeHg (of THg), rather than an inverse association, suggested that MeHg degradation was potentially less prevalent compared to the human gut. The opposite trend was observed in colon sections compared to fecal samples, suggesting that different mechanisms contributed to the proportion of MeHg (of THg) in each matrix and different organs.

### Comparison of Studies Investigating MeHg Exposure and Gut Microbiota

Several human and animal studies have investigated associations between MeHg exposure and gut microbiota communities, and results have varied between studies. For example, among 179 6-week-old infants enrolled in the New Hampshire Birth Cohort Study, exposure to 16 trace elements, including THg, was assessed in infant toenails, and the infant gut microbiota was determined using 16S gene profiling (Laue et al., 2020). However, there were no statistically significant associations between toenail THg and ASVs in adjusted regression models (Laue et al., 2020). Among two cohorts of pregnant mothers, fecal MeHg was positively correlated with several gut microbiota taxa, although specific taxa differed between both cohorts, and associations differed longitudinally between early and late gestation (*n* = 17, Rothenberg et al., 2016; *n* = 52, Rothenberg et al., 2019).

In studies using experimental animals, MeHg supplementation altered the community structure of the gut microbiota; however, trends for specific taxa were not consistent. For example, among fathead minnows (*Pimephales promelas*), which were fed control (0.02 μg/g), low MeHg (0.87 μg/g), or high MeHg (5.5 μg/g) diets (*n* = 5 tanks/treatment), the relative abundances of 18 taxa differed between the three groups (Bridges et al., 2018). Male Sprague–Dawley rats were administered with MeHg orally (10 μg MeHg/kg bw); *n* = 3 rats/treatment) (Lin et al., 2020). This MeHg dose was 100 times higher than the National Research Council reference level for MeHg (0.1 μg MeHg/kg bw) (National Research Council, 2000), and as such, several gut microbiota taxa at the family level were enriched or depleted. In mice orally fed MeHg for 7 days (3.1 μg MeHg/kg bw per day; *n* = 5 mice/treatment), several gut microbiota genera/species were more enriched (or depleted) compared to controls (Zhang et al., 2019). In these studies, MeHg exposure was associated with enrichment or depletion of gut taxa; however, specific taxa differed.

In the present study, associations were observed (positive and negative) between fecal MeHg and gut microbiota taxa (Table 4, Figure 3, and Supplementary Tables 6, 7), which differed from those noted in prior studies (Rothenberg et al., 2016, 2019; Bridges et al., 2018; Zhang et al., 2019; Lin et al., 2020). The results in both human and animal studies suggest that the direction (and strength) of the association between gut microbiota taxa and MeHg depends potentially on several factors, such as diet, MeHg dose, timing of exposure, and co-exposure to other contaminants or nutrients (Rothenberg et al., 2016, 2019; Bridges et al., 2018; Guo et al., 2018; Zhang et al., 2019; Laue et al., 2020; Lin et al., 2020).

### Potential Hg Methylation in the Walrus Gut

In walruses, fecal MeHg and %MeHg (of THg) were both positively correlated with unclassified genera of the Oscillospirales order. Members of the genus *Oscillibacter* (order: Oscillospirales) have been observed in other marine mammals, such as seals from the Arctic and Antarctic (Glad et al., 2010; Nelson et al., 2013). It is unclear whether positive associations between fecal MeHg and %MeHg (of THg) and members of the Oscillospirales order potentially reflected microbial IHg methylation. Parks et al. (2013) identified the gene cluster (*hgcAB*), which is essential for microbial IHg methylation, including a putative corrinoid protein that transfers a methyl group to Hg (HgcA) and a ferredoxin protein that performs corrinoid reduction (HgcB). Using the JGI Integrated Microbial Genomes and Microbiomes database (Chen et al., 2020), we searched for bacteria containing homologs of the *hgcAB* gene cluster. However, no members of the Oscillospirales order possessed homologs of this gene cluster. Our results suggested that the source of MeHg in fecal samples was potentially from diet, rather than microbial IHg methylation, which would be consistent with prior studies among human populations (Podar et al., 2015; Rothenberg et al., 2016). However, it is important to note the Oscillospirales order is poorly studied, and it is possible that they are not currently well-represented in reference datasets.

### MeHg Intake and Excretion in Walruses and Humans

Adult male Pacific walruses weigh up to 2,000 kg and eat approximately 2–3% of their body weight per day (Kastelein et al., 2000). Nevertheless, the range of fecal Hg species among walruses was comparable to values reported among Japanese men (*n* = 4; Ishihara, 2000) and adults in Rochester, NY (*n* = 33; Caito et al., 2018; *n* = 8, Rand et al., 2016) (Table 5 and Supplementary Table 12). For example, the median THg concentration was 200 ng/g in walruses and ranged from 130 to 270 ng/g in human studies (Ishihara, 2000; Rand et al., 2016; Caito et al., 2018). The median MeHg concentration was 4.7 ng/g in walruses, which was within the same range of values among adults in Rochester, NY (MeHg range: 0–5.9 ng/g) (Rand et al., 2016; Caito et al., 2018), but was lower compared to Japanese adults (MeHg range: 21–41 ng/g) (Ishihara, 2000).

**TABLE 5:** Comparison of fecal (or colon) mercury concentrations between walruses and humans.

#	Sample	Matrix	Location	Sample size (n)	THg (ng/g dw) Median (range)	MeHg (ng/g dw) Median (range)	%MeHg (of THg) Median (range)	References
1	Walrus	Fecal	St. Lawrence Island, Alaska, United States	16	200 (72, 650)	4.7 (0.96, 70)	2.5 (0.46, 30)	This study
	Walrus	Colon	St. Lawrence Island, Alaska, United States	16	28 (15, 95)	7.8 (3.3, 57)	26 (16, 60)	
2	Adult men^*a,b*^	Fecal	Japan	4 4	270 (260–290) 190 (120–250)	41 (15–46) 21 (12–35)	17 (5.3, 29) 12 (8.4–32)	Ishihara (2000)
3	Adult men and women^*a,c*^	Fecal	Rochester, New York, United States	8 8	160 (95, 390) 170 (33, 600)	0^*d*^ (0, 63) 5.9^*d*^ (0, 80)	0 (0, 42) 5.9 (0, 23)	Rand et al., 2016
4	Adult men and women^*a*^	Fecal	Rochester, New York, United States	33	130 (47–270)	2.8^*d*^ (0, 54)	2.3 (0, 71)	Caito et al., 2018
5	Pregnant mothers, Late gestation^*e*^	Fecal	Greenville, South Carolina, United States	17	30 (2.1–810)	0.060 (0.0025–0.39)	0.12 (0.0058, 5.8)	Rothenberg et al., 2016
6	Pregnant mothers, Early gestation^*f*^	Fecal	Charleston, South Carolina, United States	28	28 (1.1, 1,400)	0.035 (0.0029, 2.1)	0.12 (0.00031, 8.9)	Rothenberg et al., 2019
	Pregnant mothers, Late gestation^*f*^	Fecal	Charleston, South Carolina, United States	24	19 (1.0, 400)	0.0093 (0.0013, 1.7)	0.050 (0.0025, 2.0)	
	Neonates	Meconium	Charleston, South Carolina, United States	14–17^*g*^	3.9 (0.64, 11)	0.0078 (<MDL, 0.078)	0.34 (0.055, 0.84)	

*dw, dry weight; MDL, method detection level; MeHg, methylmercury; THg, total mercury. ^*a*^THg and MeHg concentrations were reported in wet weight (Ishihara, 2000; Rand et al., 2016; Caito et al., 2018). Using an average wet/dry ratio for human fecal samples (4.18 unitless) (Rothenberg et al., 2019), the THg and MeHg concentrations were converted from wet weight to dry weight (see Supplementary Table 12 for THg and MeHg concentrations in wet weight). ^*b*^Samples were collected twice from the participants, in August 1997 and June 1998 (Ishihara, 2000). ^*c*^Samples were collected twice from eight participants, after trial 1 and after trial 2 (Rand et al., 2016). ^*d*^MeHg was estimated by subtraction (=total mercury - inorganic mercury). ^*e*^Late gestation: 36–39 weeks of gestation (Rothenberg et al., 2016). ^*f*^Early gestation: 8.3–17 weeks of gestation; late gestation: 27–36 weeks of gestation (Rothenberg et al., 2019). ^*g*^Sample size: THg (*n* = 14), MeHg (*n* = 17), and %MeHg (of THg) (*n* = 14) (Rothenberg et al., 2019).*

One potential explanation concerns the different trophic levels of seafood or marine prey ingested. The Pacific walrus typically feeds on lower-trophic-level clams and other bivalves (Sheffield and Grebmeier, 2009), while participants in human studies ingested higher-trophic-level seafood, including tuna steaks (Ishihara, 2000; Rand et al., 2016; Caito et al., 2018). Due to biomagnification of MeHg in the aquatic food web, concentrations of MeHg are much higher in tuna compared to bivalves (U.S. Food and Drug Administration, 2014). For example, a 1,226-kg male Atlantic walrus (*Odobenus rosmarus rosmarus*) ingested 57 kg/day of bivalves (95% confidence interval: 41–72 kg/day) (Born et al., 2003). Using the median THg concentration in clams (0.002 μg/g wet weight) (U.S. Food and Drug Administration, 2014), the Atlantic walrus potentially ingested 798 μg Hg/week (95% confidence interval: 574–1,000 μg Hg/week). In addition to bivalves, Pacific walruses also occasionally ingest higher-trophic-level prey, including forage fish, seabirds and other pinniped species, such as ice seals (Sheffield and Grebmeier, 2009; Bowles and Trites, 2013; Seymour et al., 2014; Maniscalco et al., 2020; Sonsthagen et al., 2020). Nevertheless, the range of values estimated for Pacific walruses was still 2.8–14.5 times higher than the weekly Hg ingestion rate reported for adults in Rochester, NY (144–360 μg Hg/week, Caito et al., 2018; 100–150 μg Hg/week, Rand et al., 2016), and in Japan (70 μg Hg/week) (Ishihara, 2000). Given the amount of marine prey ingested by walruses, our results suggest that walruses absorb a higher percentage of MeHg into their tissues and excrete less MeHg compared to some human studies (Ishihara, 2000; Rand et al., 2016; Caito et al., 2018).

### Limitations

Although our study has many strengths, there are some limitations to note. First, this was a cross-sectional study, and it was not possible to determine the direction of causality between gut microbiota taxa and Hg species in fecal and colon samples. Although microbial degradation of MeHg was potentially important, it was also possible that MeHg impacted the community structure of the gut microbiota (Bridges et al., 2018; Zhang et al., 2019; Lin et al., 2020). Positive associations between gut microbiota alpha diversity indices and MeHg and %MeHg (of THg) suggested that MeHg potentially impacted the gut microbiota, although the mechanisms were uncertain. In addition, our sample size was limited; however, our results suggested that associations between MeHg and gut microbiota differed from IHg, which was consistent with prior studies in humans (Rothenberg et al., 2016, 2019). As noted in the section “Materials and Methods,” we used the Earth Microbiome Project-modified primer pair 515F-806R, which does not give a full picture of the archaeal members of the microbiome (Pausan et al., 2019). While *hgcAB* has been reported in Bacteria, it has also been found in multiple Archaea (including the gut commensal, *M. luminyensis*) (Parks et al., 2013), and thus some potentially important microbes, relevant to Hg metabolism, were not likely represented in the sequence dataset. There was an insufficient mass of remaining fecal sample for analyses of the *hgcAB* gene cluster (Gionfriddo et al., 2020) or MeHg demethylation (Lu et al., 2016); however, these assays will be included in future studies.

## Conclusion

In conclusion, variability in MeHg metabolism was observed in Pacific walruses, which was not likely due to differences in prey trophic level. The source of MeHg in the gut was potentially from consumption of marine prey, rather than microbial IHg methylation within the digestive tract. Despite the amount of marine prey ingested by walruses, less MeHg appeared to be excreted by walruses compared to some human studies. Moreover, fecal MeHg and %MeHg (of THg) were weakly positively correlated in walruses, which differed from human studies, and suggested that MeHg degradation was potentially less prevalent in the walrus gut. Accumulation of MeHg in colon tissue possibly reflected alterations in the mucosa. In paired fecal–colon samples from walruses, MeHg was more concentrated in the colon compared to fecal samples, suggesting that MeHg was able to pass through the colon tissue into systemic circulation. Thus, MeHg metabolism in the Pacific walrus may contribute to relatively higher MeHg accumulation in walrus tissues, which is important for the Arctic indigenous communities who ingest walruses and other marine foods as part of their diet (Arctic Monitoring and Assessment Program (AMAP), 2011).

## Data Availability Statement

The 16S rRNA gene sequencing data generated for this study have been deposited to the National Center for Biotechnology Information (NCBI) under accession number PRJNA705500.

## Ethics Statement

Ethical review and approval was not required for the animal study because the Oregon State University IACUC issued a waiver. The samples were collected by Alaskan hunters as part of the Alaskan Subsistence Harvest, through the US Fish and Wildlife Service.

## Author Contributions

HB, SS, and BB conceived and designed the study and collected and analyzed the data. DS, BR, CC, LC, and SR contributed to the data collection and analysis. SR wrote the manuscript. All the authors contributed to manuscript revision and read and approved the manuscript.

## Conflict of Interest

The authors declare that the research was conducted in the absence of any commercial or financial relationships that could be construed as a potential conflict of interest.

## Footnotes

1 https://www.protocols.io/view/earth-microbiome-project-emp-high-throughput-htp-d-pdmdi46.

2 https://earthmicrobiome.org/protocols-and-standards/16s/.
