# Supplementary material for: Fecal Methylmercury Correlates With Gut Microbiota Taxa in Pacific Walruses (Odobenus rosmarus divergens)
Source: Front Microbiol. 2021 Jun 9;12:648685. doi: 10.3389/fmicb.2021.648685 (PMC8220164; doi:10.3389/fmicb.2021.648685)
Supplement: Supplementary file 1 [file Data_Sheet_1.docx]

***SUPPLEMENTARY MATERIAL***

**Supplementary Table S1**. Quality assurance and quality control for mercury analyses in stool and colon samples.

|  | **% Recovery of reference materials or spikes** | | |  |
| --- | --- | --- | --- | --- |
|  | **Tort2**  **(lobster)**  **Mean ± SD**  **(n)** | **IAEA-086**  **(hair)**  **Mean ± SD**  **(n)** | **Matrix spike**  **Mean ± SD**  **(n)** | **%RSD**  **Mean ± SD**  **(n)** |
| **THg** | 99 ± 9.1  (n=6) | 88 ± 3.5  (n=2) | NA | 20 ± 14  (n=23) |
| **MeHg** | 96 ± 24^a^  (n=9) | NA | 82 ± 22  (n=4) | 17 ± 18^b^  (n=7) |

IAEA (International Atomic Energy Agency), MeHg (methylmercury), n (sample size), NA (not applicable), %RSD (relative standard deviation = 100*sd/mean), SD (standard deviation), THg (total mercury)

^a^Tort2 MeHg was analyzed after digestion in 5 mL of 25% (w/v) potassium hydroxide-methanol in a 70 ^o^C incubator for three hours. Digests were analyzed following EPA Method 1630 (2001), as described in the text.

^b^When one value was removed, the %RSD average ± SD was 10 ± 7.5% (n=6).

**Supplementary information for DADA2 pipeline**

The raw sequence data were processed through the DADA2 pipeline using the following trimming parameters: truncLen=c(200,180), trimLeft = 20, maxN=0, maxEE=c(2,2), truncQ=2. Default parameters were used for estimating error parameters using learnErrors(), and chimeras were removed using removeBimeraDenova (method = “consensus”).

**Supplementary Table S2**. This table includes tracking throughout the DADA2 pipeline, as follows: input is the number of reads, filtered is the number of filtered reads, denoisedF is the number of denoised forward reads, denoisedR is the number of denoised reverse reads, and the total number of final amplicon sequence variants (ASVs) is in the nonchim column (n=16 walruses).

|  | **input** | **filtered** | **denoisedF** | **denoisedR** | **merged** | **nonchim** |
| --- | --- | --- | --- | --- | --- | --- |
| **lane1-s001-index-CCTCGCATGACC-S190023-16s** | 138456 | 124591 | 124331 | 124286 | 122669 | 120903 |
| **lane1-s002-index-CGCGCAAGTATT-S190039-16s** | 127531 | 123331 | 123060 | 123078 | 120754 | 113291 |
| **lane1-s005-index-ACGGCGTTATGT-S190025-16s** | 124763 | 120696 | 120071 | 120384 | 117551 | 109670 |
| **lane1-s006-index-CATTTGACGACG-S190048-16s** | 140413 | 133120 | 132775 | 132703 | 128611 | 106324 |
| **lane1-s008-index-GAGAAGCTTATA-G190023-16s** | 135679 | 128259 | 127802 | 127853 | 124381 | 116390 |
| **lane1-s009-index-GGCGTAACGGCA-G190058-16s** | 142089 | 134057 | 133560 | 133049 | 126004 | 103811 |
| **lane1-s010-index-AATACAGACCTG-S190052-16s** | 127131 | 120873 | 120541 | 120505 | 118193 | 107142 |
| **lane1-s011-index-GATCTAATCGAG-S190047-16s** | 149003 | 140291 | 139925 | 139915 | 135707 | 121701 |
| **lane1-s012-index-CCGCAGCCGCAG-G190054-16s** | 121401 | 105935 | 105332 | 105056 | 97231 | 74403 |
| **lane1-s014-index-ACTAAGTACCCG-S190060-16s** | 123636 | 116896 | 116498 | 116480 | 112422 | 92821 |
| **lane1-s015-index-CTCAGCGGGACG-S190010-16s** | 107325 | 101583 | 101279 | 101301 | 98538 | 93076 |
| **lane1-s016-index-GTTAACTTACTA-S190021-16s** | 136792 | 125549 | 124894 | 124781 | 118632 | 98176 |
| **lane1-s017-index-GCGAGGAAGTCC-S190029-16s** | 167369 | 142807 | 142072 | 142006 | 137476 | 129309 |
| **lane1-s018-index-GGACAAGTGCGA-G190024-16s** | 147491 | 140223 | 139304 | 139458 | 131622 | 106559 |
| **lane1-s020-index-TGGAGCCTTGTC-S190030-16s** | 146521 | 125880 | 125226 | 124687 | 120225 | 114500 |
| **lane1-s023-index-ATGCCTCGTAAG-SterileWater-16s** | 197186 | 121487 | 120716 | 120606 | 115104 | 111591 |
| **lane1-s028-index-TTACTTATCCGA-S190053-16s** | 121523 | 111115 | 110440 | 110389 | 106100 | 96719 |

**Supplementary Table S3. Twenty-four taxa included in Maaslin2 analyses.**

**Supplementary Table S4. Results from Maaslin2 for fecal log_10_ total mercury (THg) versus 24 taxa.**

**Supplementary Table S5. Results from Maaslin2 for fecal log_10_ inorganic mercury (IHg) versus 24 taxa.**

**Supplementary Table S6. Results from Maaslin2 for fecal log_10_ methylmercury (MeHg) versus 24 taxa.**

**Supplementary Table S7. Results from Maaslin2 for fecal log_10_ %methylmercury (MeHg) (of total mercury) versus 24 taxa.**

**Supplementary Table S8. Results from Maaslin2 for colon log_10_ total mercury (THg) versus 24 taxa.**

**Supplementary Table S9. Results from Maaslin2 for colon log_10_ inorganic mercury (IHg) versus 24 taxa.**

**Supplementary Table S10. Results from Maaslin2 for colon log_10_ methylmercury (MeHg) versus 24 taxa.**

**Supplementary Table S11. Results from Maaslin2 for colon log_10_ %methylmercury (MeHg) (of total mercury) versus 24 taxa.**

**Supplementary Table S12**. Mercury concentrations in fecal samples for studies, in which stool mercury concentrations were reported in wet weight (see Table 5 for estimated dry weight concentrations).

| **#** | **Sample** | **Matrix** | **Location** | **Sample size**  **(n)** | **THg**  **Median**  **(Range)**  **(ng/g ww)** | **MeHg**  **Median**  **(Range)**  **(ng/g ww)** | **%MeHg**  **(of THg)**  **Median**  **(Range)** | **Reference** |
| --- | --- | --- | --- | --- | --- | --- | --- | --- |
| 2 | Adult men^1^ | Fecal | Japan | 4  4 | 65  (62-69)  44  (28-60) | 9.9  (3.6-11)  5.0  (2.8-8.4) | 17  (5.3, 29)  12  (8.4-32) | Ishihara et al., 2000 |
| 3 | Adult men and women^2^ | Fecal | Rochester, New York, USA | 8  8 | 37.8  (22.8, 93.1)  41.75  (8, 144.5) | 0^3^  (0, 15.1)  1.4^3^  (0, 19.1) | 0  (0, 42)  5.9  (0, 23) | Rand et al., 2016 |
| 4 | Adult men and women | Fecal | Rochester, New York, USA | 33 | 30.4  (11.2-63.5) | 0.66^3^  (0, 13) | 2.3  (0, 71) | Caito et al., 2018 |

MeHg (methylmercury), n (sample size), THg (total mercury), ww (wet weight)

^1^Samples were collected twice from 4 participants, in August 1997 and June 1998 (Ishihara, 2000)

^2^Samples were collected twice from 8 participants, after Trial 1 and after Trial 2 (Rand et al., 2016)

^3^MeHg estimated by subtraction (=total mercury-inorganic mercury)


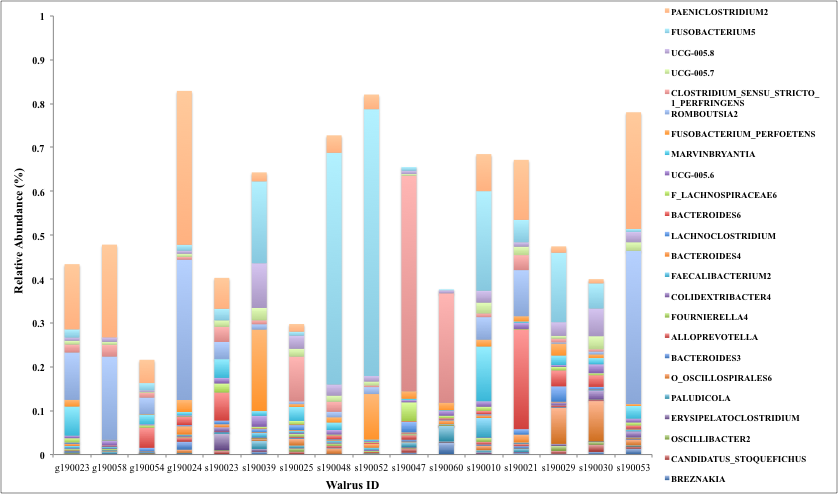


**Supplementary Figure S1.** Relative abundance (%) of the 24 most prevalent taxa (n=16 walruses). All walruses were from St. Lawrence Island, Alaska, USA, including four from the Native Village of Gambell (with id's beginning with "g"), and 12 from the Native Village of Savoonga (with id's beginning with "s"). See Supplementary Table 3 for the 24 taxa, including phylum, class, order, family, and genus.

**Supplementary References**

Caito, S.W., Jackson, B.P., Punshon, T., Scrimale, T., Grier, A., Gill, S.R., et al. (2018). Variation in methylmercury metabolism and elimination status in humans following fish consumption. Toxicol. Sci. 161, 443-453.

Ishihara, N. (2000). Excretion of methyl mercury in human feces. Arch. Environ. Health. 55, 44-47.

Rand, M.D., Vorojeikina, D., van Wijngaarden, E., Jackson, B.P., Scrimale, T., Zareba, G., et al. (2016). Methods for individualized determination of methylmercury elimination rate and de-methylation status in humans following fish consumption. Toxicol. Sci. 149, 385-395.

U.S. Environmental Protection Agency. (2001). Method 1630, Methyl Mercury in Water by Distillation, Aqueous Ethylation, Purge and Trap, and CVAFS. EPA 821-R-01-020. Washington, D.C.: U.S. Environmental Protection Agency
